# *VEGFA* GENE variation influences hallucinations and frontotemporal morphology in psychotic disorders: a B-SNIP study

**DOI:** 10.1038/s41398-018-0271-y

**Published:** 2018-10-11

**Authors:** Paulo Lizano, Olivia Lutz, George Ling, Jaya Padmanabhan, Neeraj Tandon, John Sweeney, Carol Tamminga, Godfrey Pearlson, Gualberto Ruaño, Mohan Kocherla, Andreas Windemuth, Brett Clementz, Elliot Gershon, Matcheri Keshavan

**Affiliations:** 1 Department of Psychiatry, Beth Israel Deaconess Medical Center, Boston, MA USA; 2 Department of Psychiatry, Harvard Medical School, Boston, MA USA; 3 Department of Psychiatry, University of Cincinnati Medical Center, Cincinnati, OH USA; 4 University of Texas Southwestern Medical Center, Dallas, TX USA; 5 Hartford Hospital, Yale School of Medicine, Hartford, CT USA; 6 Genomas Inc., Hartford, CT USA; 7 Department of Psychology, University of Georgia, Athens, GA USA; 8 Department of Psychiatry and Behavioral Neurosciences, University of Chicago, Chicago, IL USA

## Abstract

Vascular endothelial growth factor A (*VEGFA*) dysfunction may contribute to a number of pathological processes that characterize psychotic disorders. However, the influence of *VEGFA* gene variants on clinical and neuroimaging phenotypes in psychotic disorders has yet to be shown. In the present study, we examined whether different *VEGFA* gene variants influence psychosis risk, symptom severity, cognition, and brain volume. The study group included 480 probands (Bipolar I disorder with psychosis, *n* = 205; Schizoaffective disorder, *n* = 112; Schizophrenia, *n* = 163) and 126 healthy controls that were recruited across six sites in the B-SNIP consortium. *VEGFA* variants identified for analysis (rs699947, rs833070, and rs2146323) were quantified via SNP chip array. We assessed symptoms and cognition using standardized clinical and neuropsychological batteries. The dorsolateral prefrontal cortex (DLPFC), medial temporal lobe, and hippocampal volumes were quantified using FreeSurfer. In our sample, *VEGFA* rs2146323 A- carriers showed reduced odds of being a proband (*p* = 0.037, OR = 0.65, 95% CI = 0.43–0.98) compared to noncarriers, but not for rs699947 or rs833070. In probands, rs2146323 A- carriers demonstrated fewer hallucinations (*p* = 0.035, Cohen’s *d* = 0.194), as well as significantly greater DLPFC (*p* < 0.05, Cohen’s *d* = −0.21) and parahippocampal volumes (*p* < 0.01, Cohen’s *d* = −0.27). No clinical or neuroimaging associations were identified for rs699947 or rs833070. In general, we found that the three SNPs exhibited several significant negative relationships between psychosis symptoms and brain structure. In the probands and control groups, positive relationships were identified between several cognitive and brain volume measures. The findings suggest *VEGFA* effects in the DLPFC and hippocampus found in animals may also extend to humans. *VEGFA* variations may have important implications in identifying dimensional moderators of function that could be targeted through *VEGFA-*mediated interventions.

## Introduction

Vascular endothelial growth factor A (*VEGFA*) dysfunction may contribute to a number of pathological processes that characterize psychotic disorders. *VEGFA* is an essential molecule for angiogenesis, neurogenesis, and brain plasticity and is distinct from other neurotrophic factors in its major angiogenic function^1,2^. *VEGFA* can promote proliferation of endothelial cells, neural stem cells, neurons, and mature astrocytes^1^. In the adult rodent brain, an enriched environment or overexpression of *VEGFA* can stimulate neovascularization, hippocampal neurogenesis, and improve cognitive performance, whereas blocking endogenous *VEGFA* function impairs the angiogenic, neurogenic, and cognitive response to neuro-enhancing stimuli^1–6^.

In schizophrenia, altered *VEGFA* signaling has been implicated via transciptomic and epigenomic analysis of prefrontal cortical post mortem samples^5,7–11^. Imaging genetic studies of *VEGFA* single nucleotide polymorphisms (SNPs) in healthy subjects have demonstrated that minor allele carriers for rs833070 and rs2146323 have smaller hippocampal volumes and rs699947 minor allele carriers have reduced total gray and white matter volume^12,13^. These polymorphisms were also significantly different (*p* < 3e−3, OR = 0.97 to 1.04, comparing schizophrenia vs. controls) in the Psychiatric Genome Consortium (PGC) SZ working group analysis, but this did not meet genome-wide level of significance at 5 < 10e−8^14^. It is well established that antipsychotic treatment results in changes in VEGFA levels that are related to the Positive and Negative Symptom Scale (PANSS) positive symptoms but not negative symptoms. Additionally, we have identified robust cognitive deficits and smaller medial temporal lobe (MTL) structures in psychosis probands compared to controls, and these abnormalities correlated with cognition and psychosis severity^15–17^. However, there are no studies to date examining the effects of these *VEGFA* polymorphisms on clinical and structural neuroimaging phenotypes in psychotic disorders.

This study was based on the cross-sectional Bipolar-Schizophrenia Network on Intermediate Phenotypes (B-SNIP) consortium, which assessed clinical, cognitive, and neurobiological phenotypes across the psychotic spectrum. We took an integrated genetic and phenotype approach to (1) investigate the disease risk associated with rs699947, rs833070, and rs2146323, (2) determine the effects of these genetic variants on positive symptoms, memory, and executive function, and (3) evaluate their influence on DLPFC and MTL structure as well as hippocampal morphology.

## Materials and methods

### Study participants

The study group included 480 probands (*n* = 205 Bipolar I with psychosis, BPP; *n* = 112 Schizoaffective disorder, SAD; *n* = 163 Schizophrenia, SZ) and 126 healthy controls (HC) that were recruited as part of the B-SNIP multisite study at Harvard University, Wayne State University, Maryland Psychiatric Research Center, University of Texas Southwestern Medical Center at Dallas, Institute of Living/Yale University, and University of Illinois at Chicago. Detailed sample information is provided elsewhere and participants provided informed consent^18^.

All subjects met the following inclusion criteria: ages 15–65, sufficient English proficiency, no history of neurological disorders or head injuries, no history of substance abuse in the last month or substance dependence in the last 6 months and a negative urine toxicology screen on the day of testing. Healthy controls also met criteria for no personal history of recurrent mood disorders, no personal or family history of psychotic disorders, no history of substance dependence and no history of any significant cluster A axis II personality features within the structured interview for DSM-IV-TR Personality. All sites used identical recruitment, clinical and diagnostic approaches that were approved by respective institutional review boards^18^.

Diagnoses were based on the Structured Clinical Interview for DSM-IV-TR (SCID-IV). Subjects received consensus diagnosis using information from clinical interviews, medical records, and family members. Symptom ratings were completed using PANSS. We focused our analysis on total positive symptom, delusion, and hallucination scores since they have been previously associated with *VEGFA*^10,19,20^. Cognition was evaluated using the Brief Assessment of Cognition (BACS) battery and a full presentation of these data is available in ref. ^15^. The Verbal Memory, Digit Sequence, Symbol Coding, and Tower of London subscales from BACS were used to assess verbal declarative memory, working memory, processing speed, and reasoning/problem solving, respectively. All scores were adjusted for age and sex and transformed to z-scores with a range of ±4.0.

### Structural T1 imaging

Structural T1-weighted images were acquired across six sites: Boston (3.0 T, GE Signa), Detroit (3.0 T, Siemens Allegra), Baltimore (3.0 T, Siemens Trio trim), Hartford (3.0 T, Siemens Allegra), Dallas (3.0 T, Philips), and Chicago (3.0 T, GE Signa). Structural T1 MPRAGE isovoxel scans (TR = 6.7 ms, TE = 3.1 ms, 8° flip angle, 256 × 240 matrix size, total scan duratio*n* = 5:26 min, 170 sagittal slices, 1 mm slice thickness, 1 × 1 × 1.2 mm^3^ voxel resolution) were acquired with the Alzheimer’s Disease Neuroimaging Initiative (ADNI) protocol (http://adni.loni.usc.edu).

Scans were assessed for motion and scanner artifacts and 51 subjects failed the first level of quality control. The passing scans were processed using FreeSurfer 5.1 ^21^ and were run through a first-level auto-reconstruction. Trained raters removed dura, sinuses, and vessels from the skull-stripped images that could interfere with segmentation and all raters demonstrated a 95% inter-rater reliability. The images were run through a second- and third-level auto-reconstruction to extract volume measures. The gray matter volume for the dorsolateral prefrontal cortex (DLPFC) (calculated by summating the caudal and rostral middle frontal regions), entorhinal, parahippocampal, and hippocampus were extracted from the DKT atlas and bilateral values were summated.

### SNP selection and genotyping

The human *VEGFA* gene is located on chromosome 6p12 (43,768,581–43,788,115) (GRCh38.p7; GCF_000001405.33) and includes eight exons separated by seven introns. We selected rs833070 (intron), rs2146323 (intron), and rs699947 (promoter) for tag SNPs that influence brain morphology in healthy individuals^12,22^, confer schizophrenia risk^14^, and have minor allele frequencies (MAF) ≥ 0.05 in the HapMap-CEU population. More specifically, we examined publicly available databases, reviewed the literature on *VEGFA* polymorphisms in schizophrenia, and selected high-risk variants from NCBI, and the subsequent list was cross-referenced with the PGC schizophrenia working group findings which led to a selection of the three above-mentioned SNPs.

Blood samples were drawn after structural MRI scanning. Subjects’ DNA was genotyped using an Illumina’s Human Omni1-Quad Bead Chip for 1,140,419 common SNPs at Genomas Inc (Hartford Hospital, CT) and detailed information can be found at Meda et al.^23^. Genotyped data were preprocessed in PLINK v1.07 (http://pngu.mgh.harvard.edu/purcell/plink/) following a published guideline for SNP selection in candidate genes/regions^24^. As an exploratory aim, we evaluated SNP distribution across biotypes generated by Clementz et al. using electrophysiology, eye tracking, and cognitive measures in the B-SNIP study with biotype 1 being the most compromised and 3 the least^25^.

### Statistical analyses

A one-way analysis of variance and *χ*^2^ tests were used to test for between-group differences in demographic and clinical variables. We performed the analysis in the proband (combined groups consisting of SZ, SAD, BPP) vs. control groups to enhance the study power and to demonstrate that *VEGFA* genotype to phenotype features cross diagnostic boundaries. Genotype and allele frequencies were calculated by the gene-counting method and each polymorphism was tested for Hardy−Weinberg equilibrium using *χ*^2^ goodness-of-fit tests using the R package SNPassoc (http://www.creal.cat/jrgonzalez/ software.htm). The association between target SNPs and the risk of being a proband under two inheritance models, codominant and dominant models, was performed using logistic regression, adjusting for age, sex, and ethnicity factors. To validate our results, we performed a (1) site-specific analysis with sites containing a large enough sample size and (2) we created a training and test dataset with a 70/30 random split of our whole sample. All statistical tests were two-sided and *p* < 0.05 was defined as statistically significant. Bonferroni correction was applied by multiplying the *p* values by the three SNPs being examined, which were carefully selected by identifying relevant SNPs from the PGC schizophrenia and bipolar datasets, as well as two structural neuroimaging studies^12–14,26^. Subjects with imaging measures outside of four standard deviations were removed (*n* = 1) and those between three and four standard deviations were winsorized to the third standard deviation (*n* = 12). Contrasts between carriers and noncarriers within a group (HC or Probands) were run on clinical, cognitive and imaging measures using an analysis of covariance (ANCOVA) or general linear hypothesis test while controlling for age, sex, ethnicity, and site for a total of 36 comparisons (3 SNPs and 12 traits) for a Bonferroni corrected *p* value of 0.0014. In probands, partial correlations within a phenotype and genotype were run using Kendall-tau correlations, adjusting for age, sex, ethnicity, and site.

As in previous genetic neuroimaging studies^12,13,27^ we included age, sex, ethnicity, and site as covariates when examining clinical and neuroimaging variables and excluded intracranial volume (ICV) for neuroimaging analysis in our basic model. We avoided correcting for ICV since it could be related to the disease and diminish the possibility of detecting associations that differentiate cases and controls, or that are related to the evolution of the disease. We also performed an analysis covarying for duration of illness, PANSS total negative symptoms, and average chlorpromazine equivalents. Also, the genotypes were dichotomized into those homozygous for the more frequent allele and “carriers” of the less frequent allele.

### Code availability

Code is available upon request my emailing the corresponding author.

## Results

Our *VEGFA* SNP sample included 606 participants (HC, *n* = 126, Proband, *n* = 480), consisting of 303 males (HC, *n* = 53; Proband, *n* = 250) and 303 females (HC, *n* = 73; Proband, *n* = 230) (Supplementary Table S1). The mean age of our sample was 35.6 years (HC, 37.3 years ± 12.4; Proband, 35.2 years ± 12.5). The groups did not differ by age (F = 2.7; *p* = 0.10), sex (F = 3.6; *p* = 0.057) or ethnicity (F = 3.3; *p* = 0.192). However, they did show significant site (F = 13.1; *p* = 0.022) effects but no group by site interactions were observed. Sample characteristics by diagnostic group or biotype group, and polymorphism can be found in Supplementary Tables S2, S3.

### Genotype distribution

The *VEGFA* polymorphisms were in Hardy−Weinberg equilibrium in probands, HC, and in the combined group (*p* > 1e−5). The call rates were 98.5% for rs2146323, 99.9% for rs833070, and 92.5% for rs699947 in the two groups. The analysis for the three tag SNPs revealed a significant difference for rs2146323 in the distribution of the codominant (*p* = 0.005, OR = 1.47, 95% CI = 0.63–3.43) and dominant (*p* = 0.037, OR = 0.65, 95% CI = 0.43–0.98) models between probands and HC (Table 1). The significant association between rs2146323 and proband membership survived adjustment for age, sex, and ethnicity in the codominant (*p* = 0.007) and dominant model (*p* = 0.042). For rs2146323, the A- carriers had significantly lower odds of being a proband when compared to the CC individuals. No significant association was observed between the other two tag SNPs and proband membership (*p* > 0.05). We replicated our finding for rs2146323 in our site-specific analysis at our Baltimore site (HC *n* = 37, Proband *n* = 130, codominant *p* = 0.002), but not for the other three sites. We found rs833070 to be significantly different in our Detroit site (HC *n* = 20, Proband *n* = 49, codominant *p* = 0.039), but not in the other sites. We replicated our rs2146323 finding in the training set (HC *n* = 88, Proband *n* = 336, codominant *p* = 0.0143), but not in the test dataset (HC *n* = 38, Proband *n* = 144, codominant *p* = 0.513). Biotype 1 group was significantly less likely to be an A- carrier compared to the other biotype or diagnostic groups (Supplementary Table S3).

**Table 1:** Logistic regression analysis of *VEGFA* SNPs in probands and control subjects

	Genotype							Allele						
	*N* (%)			OR	95% CI	*p* value	Adj *p* value	*N* (%)		OR	95% CI	*p* value	Adj *p* value	*p* of HWE^a^
rs699947	GG	GT	TT					GG	T-					
Controls	40 (35)	49 (43)	26 (22)	0.89	0.56–1.43			40 (35)	75 (65)					0.15
Probands	169 (38)	185 (41)	92 (21)	0.84	0.48–1.46	0.80	0.79	169 (38)	277 (62)	0.87	0.57–1.34	0.54	0.56	
rs833070	GG	AG	AA					GG	A-					
Controls	37 (29)	64 (51)	25 (20)	0.67	0.43–1.05			37 (29)	89 (71)					0.78
Probands	176 (37)	204 (42)	99 (21)	0.83	0.47–1.46	0.21	0.23	176 (37)	303 (63)	0.72	0.47–1.10	0.12	0.12	
rs2146323	CC	AC	AA					CC	A-					
Controls	47 (40)	64 (54)	7 (6)	0.56	0.37–0.85			47 (40)	71 (60)					0.01
Probands	242 (51)	184 (38)	53 (11)	1.47	0.63–3.43	0.005*	0.007*	242 (51)	237 (49)	0.65	0.43–0.98	**0.037**	**0.042**	

*VEGFA* vascular endothelial growth factor A, *SNP* single nucleotide polymorphism, *N* number, *OR* odds ratio, *CI* confidence interval, *HWE* Hardy–Weinberg Equilibrium

*Survived Bonferroni correction; Adj *p* value covaried for age, sex, race

^a^HWE test was carried out in controls

The bolded values stand for signficant uncorreted p values (p < 0.05)

### Psychopathology and cognition

The PANSS scores for the probands were: positive total score = 15.2 ± 5.5; delusions score = 2.5 ± 1.4; and hallucinations score = 2.4 ± 1.5 (Supplementary Table S1). There were no significant associations between rs699947 or rs833070 and PANSS positive total, delusions, or hallucinations scores (Table 2). For rs2146323 we found that, compared with subjects homozygous for the C allele, A- carriers had significantly reduced PANSS hallucinations scores (*p* = 0.035, Cohen’s *d* = −0.194). There was no significant difference between scores for positive total or delusions and rs2146323 A- carriers (Table 2). The results were no longer significant after adding average daily chlorpromazine equivalence as a covariate.

**Table 2:** Effect sizes for rs699947, rs833070, and rs2146323 SNP effect on psychopathology and cognition in probands and control subjects

	NC						Probands					
	rs699947 (GG vs. T-)		rs833070 (GG vs. A-)		rs2146323 (CC vs. A-)		rs699947 (GG vs. T-)		rs833070 (GG vs. A-)		rs2146323 (CC vs. A-)	
Psychopathology	*p* value	Cohen’s *d*	*p* value	Cohen’s *d*	*p* value	Cohen’s *d*	*p* value	Cohen’s *d*	*p* value	Cohen’s *d*	*p* value	Cohen’s *d*
Positive total	—	—	—	—	—	—	0.873	0.015	0.728	0.032	0.396	0.079
Delusions	—	—	—	—	—	—	0.615	−0.049	0.649	−0.043	0.931	0.009
Hallucinations	—	—	—	—	—	—	0.109	0.157	0.059	0.176	**0.035**	**0.194**
Cognition	*p* value	Cohen’s *d*	*p* value	Cohen’s *d*	*p* value	Cohen’s *d*	*p* value	Cohen’s *d*	*p* value	Cohen’s *d*	*p* value	Cohen’s *d*
Composite	0.210	−0.224	0.427	−0.146	0.198	−0.249	0.727	−0.033	0.675	−0.038	0.559	−0.053
Verbal Memory	0.998	0.020	0.578	−0.106	0.234	−0.219	0.072	−0.167	0.140	−0.135	0.23	−0.109
Digit Sequence	0.213	−0.218	0.453	−0.146	0.108	−0.299	1.000	−0.007	0.904	−0.011	0.994	0.000
Tower of London	0.093	−0.326	0.371	−0.164	0.298	−0.221	0.284	−0.103	0.599	−0.049	0.741	−0.030
Symbol Coding	0.565	−0.109	0.468	−0.129	0.150	−0.269	0.637	0.046	0.649	−0.039	0.519	–0.059

Psychopathology, PANSS (Positive and Negative Syndrome Scale); Cognition, BACS (Brief Assessment of Cognition); for psychopathology measures covaried for age, sex, site, and ethnicity. Adjusted cognition measures were covaried for site and ethnicity

*HC* healthy control

The bolded values stand for signficant uncorreted p values (p < 0.05)

The proband and control groups differed by Verbal Memory (F = 56; *p* = < 0.001), Digit Sequence (F = 67; *p* = < 0.001), Tower of London (F = 19; *p* = < 0.001), and Symbol Coding (F = 120; *p* = < 0.001) (Supplementary Table S1). There were no SNP effects on cognition in either the control or proband group (Table 2).

### Neuroimaging data

No significant differences for ICV (F = 0.94, *p* = 0.33) existed between probands and HC (Supplementary Table S1). There were significant smaller volumes in probands compared to HC for the DLPFC, hippocampus, parahippocampus, and entorhinal cortex (data not shown).

**Fig. 1:**
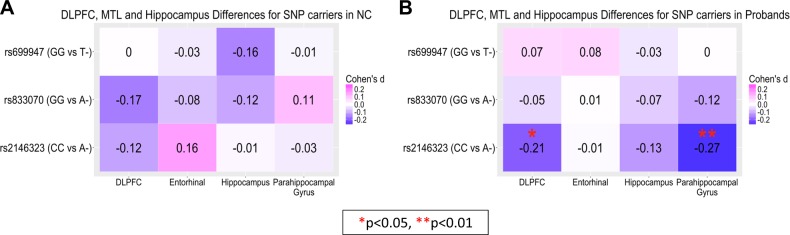
Effect sizes for rs699947, rs833070, and rs2146323 SNP effect on dorsolateral prefrontal cortex (DLPFC), entorhinal cortex, parahippocampal gyrus, and hippocapus in **a** healthy controls (NC) and **b** proband subjects

In controls, there were no significant findings identified between rs699947, rs833070, or rs2146323 and brain structure (Fig. 1). Also, there weren’t any significant differences found between brain structure and rs699947 or rs833070 in the proband group. However, for rs2146323 in probands we found that, compared with subjects homozygous for the C allele, A- carriers demonstrated greater brain volume in the DLPFC (*p* < 0.05, Cohen’s *d* = −0.21) and parahippocampal gyrus (*p* < 0.01, Cohen’s *d* = −0.27) when covaried for age, sex, ethnicity, and site, but not for the entorhinal cortex or hippocampus (Fig. 1). Both regions remain significant after adding total PANSS negative symptoms as a covariate (DLPFC, *p* = 0.038, Cohen’s *d* = −0.211; parahippocampal gyrus, *p* = 0.007, Cohen’s *d* = −0.277) but was no longer significant after covarying for total ICV (*p* > 0.05). Parahippocampal volume remained significant after adding duration of illness (*p* = 0.005, Cohen’s *d* = −0.287) and average daily chlorpromazine equivalence (*p* = 0.001, Cohen’s *d* = −0.414) as covariates.

### Clinical and BACS correlations in probands

**Fig. 2:**
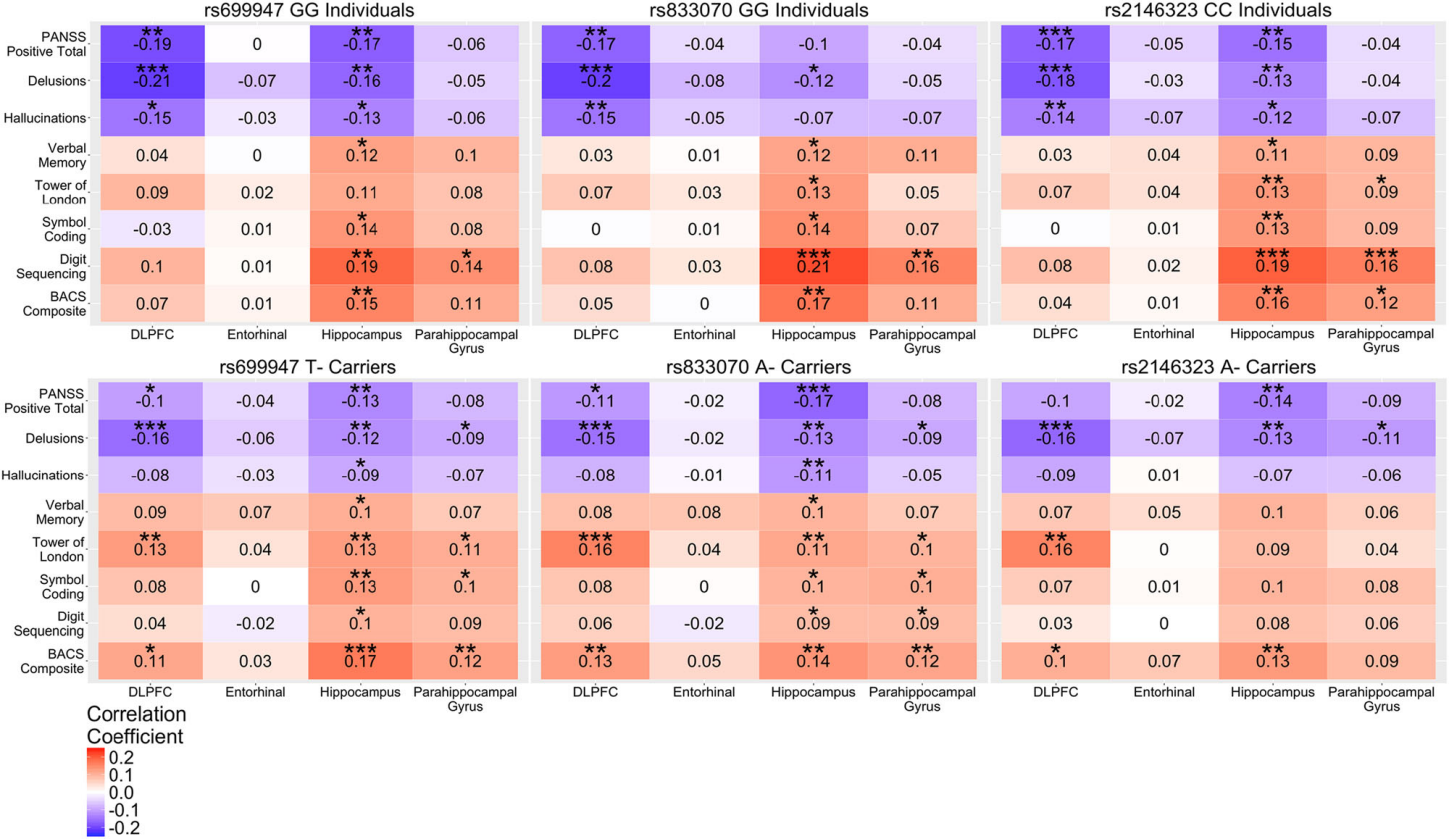
DLPFC, entorhinal, parahippocampal, and hippocampal volume correlations with symptom severity and cognition in probands by rs699947, rs833070, and rs2146323 SNP status. Values in the box indicate Kendall-tau correlations. *p<0.05; **p<0.01; ***p<0.001 (Survived Bonferroni correction)

**Fig. 3:**
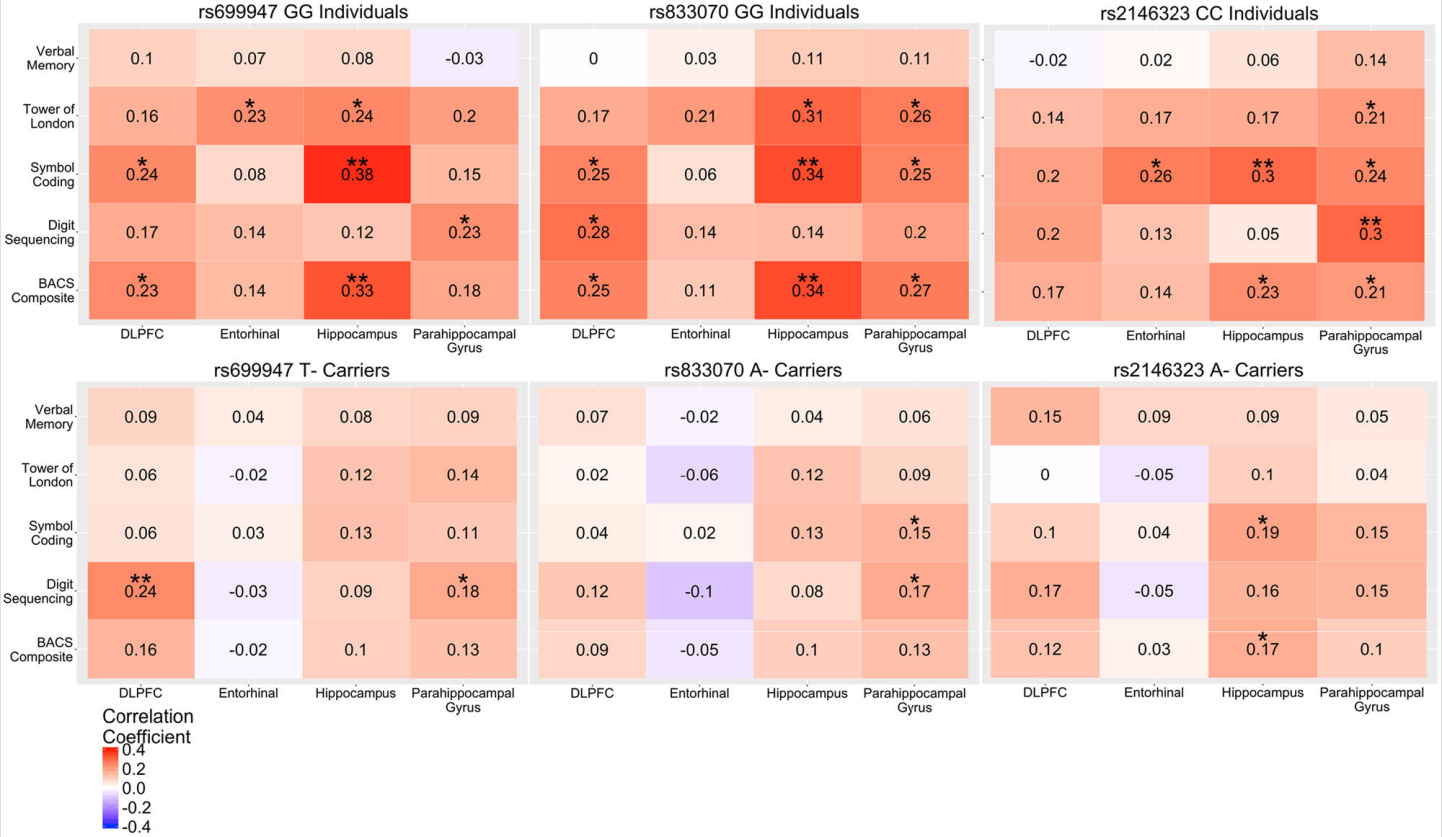
DLPFC, entorhinal, parahippocampal, and hippocampal volume correlations with cognition in healthy controls by rs699947, rs833070, and rs2146323 SNP status. Values in the box indicate Kendall-tau correlations. *p<0.05; **p<0.01; ***p<0.001 (Survived Bonferroni correction)

In probands, homozygotes for the major allele for each of the three SNPs displayed significant negative correlations (*p* < 0.05) between PANSS positive symptoms and DLPFC and hippocampal volumes, with fewer relationships observed for rs833070, and no relationship was observed between PANSS positive symptoms and entorhinal or parahippocampal volumes (Fig. 2). For cognition in probands, homozygotes for the major allele for each of the three SNPs displayed significant (*p* < 0.05) positive relationships between cognitive measures and hippocampal volume with rs246323 also having additional significant parahippocampal correlations (Fig. 2). Many of the same significant positive relationships were observed between cognition and hippocampal volume in the control sample with the addition of several significant positive correlations with the DLPFC (Fig. 3).

Similar to the above findings, minor allele carriers for each of the three SNPs showed significant negative relationships between PANSS positive symptoms and DLPFC and hippocampal volume (Fig. 2). Interestingly, rs2146323 A- carriers did not demonstrate a significant relationship between hallucination severity and hippocampal or DLPFC volume. Additionally, in probands we found that minor allele carriers for each of the three SNPs still demonstrated a significant positive relationship between cognitive domains and hippocampal volume, except for rs2146323. In controls, there were no relationships between cognition and hippocampal volume in minor allele carriers for each of the three SNPs tested, except for rs2146323, which showed significant positive relationships between symbol coding or BACS composite score and hippocampal volume (Fig. 3).

## Discussion

In summary, we found that rs2146323 A- carriers compared to CC individuals had significantly reduced odds of being a proband and that the biotype 1 group (most neurobiologically compromised) were less likely to be A- carriers. This finding was replicated in one of our sites and in our training dataset. No significant effect was noted for rs699947 or rs833070. We also demonstrated that rs2146323 A- carriers in probands were associated with reduced hallucinations and greater volume in the DLPFC and parahippocampus. We did not identify any significant clinical, cognitive, or structural associations for rs699947 or rs833070 in probands or controls. Finally, we showed that all three SNPs exhibited several significant negative relationships between psychosis symptoms and brain structure, as well as showing that in probands and control groups, positive relationships were identified between several cognitive and brain volume measures. These results support our hypothesis that *VEGFA* polymorphisms may not only play a role in reducing psychosis odds, but that rs2146323 might confer a neuroprotective effect on clinical and neuroimaging phenotypes.

In this cross-sectional study, we found that rs2146323 A- carriers were associated with a reduced odd of being a psychosis proband and we were able to replicate this finding, while rs833070 or rs699947 were not risk conferring. The PGC SZ working group identified a non-genome-wide significant associated risk for rs699947, rs833070, and rs2146323 ^14^, which further enhances the significance of our finding. However, the PGC bipolar disorder working group did not identify an associated risk for rs699947 or rs2146323 (rs833070 was not included), which included a mix of bipolar patients that were not differentiated for lifetime psychotic symptoms^26^. These discrepant results might be due to our inclusion of psychotic bipolar I disorder subjects, which is distinct from the bipolar types used in the PGC analysis. Finally, a cross-sectional genetic study of schizophrenia in a Han Chinese population showed that rs699947 nominally decreased the risk of schizophrenia in a recessive inheritance model, but they did not identify an association for rs833070, and they didn’t test for rs2146323^22^. To the best of our knowledge we are the first group to demonstrate that the *VEGFA* polymorphism, rs2146323, has a dimensional effect on psychosis membership.

The function of rs2146323 has yet to be determined, but we do know that it lies within intron 2, is located in 111-bp 5′ of exon 3, is distant from predicted transcription factor binding sites, and is not predicted to be involved in exon splicing alterations^28^. Only one study has examined the effect of the rs2146323 variant on peripheral *VEGFA* levels and they identified a significant association in their detection sample, but not in their replication sample^29^. Other studies have implicated it as a predictive maker for treatment response in improving lung function in asthmatic patients^30^, enhancing anatomic outcome in age-related macular degeneration (ARMD)^31^, and increased retinal thickness in ARMD^32^. In regards to rs699947 and rs833070, they are located on the promoter region of *VEGFA*, can result in changes in VEGF expression levels, and have been proposed to have a pharmacogenetic association in neovascular ARMD, but with inconsistent findings^32^. Our finding that rs2146323 is less frequently present in our most compromised biotype 1 group further suggests a biologically plausible mechanism involving *VEGFA*. It is possible that rs2146323 and/or rs699947 might alter *VEGFA* expression or isoform formation, which could influence brain development, neuroplasticity, and neuro-protection. Thus, these observations make rs2146323 a promising target for unveiling the mechanism of this polymorphism on *VEGFA* expression, structure, and function.

In this study, we demonstrate that rs2146323 A- carriers exert a specific reduction in PANSS hallucination subscale scores but no effect was identified for rs833070 or rs699947. There are no human or animal studies evaluating the effects of *VEGFA* polymorphisms on psychotic or cognitive phenotypes in psychotic disorders. However, our findings align with animal studies, which have shown that *VEGFA*, VEGF receptor 1 (VEGFR1/Flt-1), and VEGF receptor 2 (VEGFR2/KDR) signaling affect working memory primarily by modulating neuroplasticity at the hippocampus or through peripheral neurotrophic effects^1–6,33^. Our findings are further supported by human studies, which have demonstrated that the effects of psychotic symptoms are mixed^34^, whereas VEGR1/Flt-1 and VEGFR2/KDR dysregulation were associated with worse psychotic symptoms in a prospective familial high risk for psychosis study^35,36^, a post mortem SZ study^10^, and in case reports of systemic VEGFR1/Flt-1 administration^19,20,37^. Additionally, treatment with antipsychotic or antidepressant medications has been shown to modulate VEGF levels and is associated with improved treatment outcomes in SZ, bipolar and major depressive disorder^38–41^. Also, we would hypothesize that since rs2146323 is a potential pharmacogenetic predictor of treatment response, that the association between fewer hallucinations and rs2146323 carrier status could reflect treatment response to antipsychotics.

In our neuroimaging analysis, we found that the neuroprotective effects of rs2146323 on clinical phenotypes were also evident across the hypothesized brain regions associated with psychosis. Specifically, we found that rs2146323 A- carriers exhibited greater volume in the dorsolateral prefrontal and parahippocampal regions in probands, and this effect was specific to rs2146323 and not rs699947 or rs833070. This is the first study to research the effects of polymorphisms on brain structure in a psychotic disorder group. One healthy volunteer study assessed the effects of four variants (rs833068, rs833070, rs2146323, and rs3025020), and showed that rs2146323 and rs833070 carriers had reduced hippocampal concentrations compared to major allele carriers, but their sample was smaller than ours^12^. Additionally, we observed in probands that rs699947 T- or rs2146323 A- carriers have inverse relationships between PANSS positive symptoms and prefrontal-hippocampal structure, which is an effect also seen in major allele carriers for those same SNPs. In regards to cognition, we found that the positive relationship between BACS composite score and hippocampal volume persisted for rs2146323 A- carriers and CC individuals in the proband and control group, which suggests that more brain volume was related to better cognition and lower symptom levels. Taken together, our findings relate to animal and human studies showing that it modulates cytoarchitecture, hallucinations, and memory in psychotic disorders.

The findings suggest that the effects of prefrontal cortex and hippocampus found in animals extend to humans and further understanding of the effects of variation might have important implications for identifying a subgroup of patients with psychosis that are more vulnerable to DLPFC and hippocampal pathology, as well as those that are most likely to benefit from mediating interventions.

## Electronic supplementary material

Supplementary Table 1

Supplementary Table 2

Supplementary Table 3

Supplemental tables
