# Supplementary figures and images for: VEGFA GENE variation influences hallucinations and frontotemporal morphology in psychotic disorders: a B-SNIP study
Source: Transl Psychiatry. 2018 Oct 11;8:215. doi: 10.1038/s41398-018-0271-y (PMC6181939; doi:10.1038/s41398-018-0271-y)

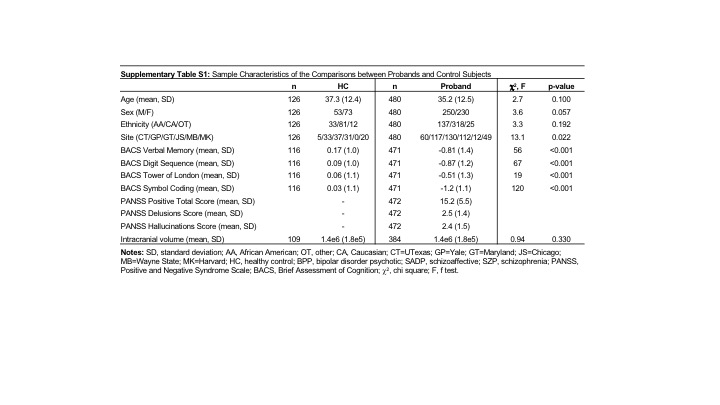

Supplement: Supplementary file 1 — Supplementary Table 1 [file 41398_2018_271_MOESM1_ESM.jpg]

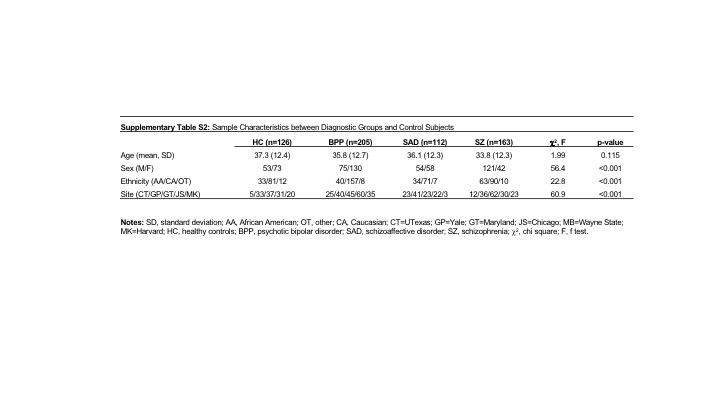

Supplement: Supplementary file 2 — Supplementary Table 2 [file 41398_2018_271_MOESM2_ESM.jpg]

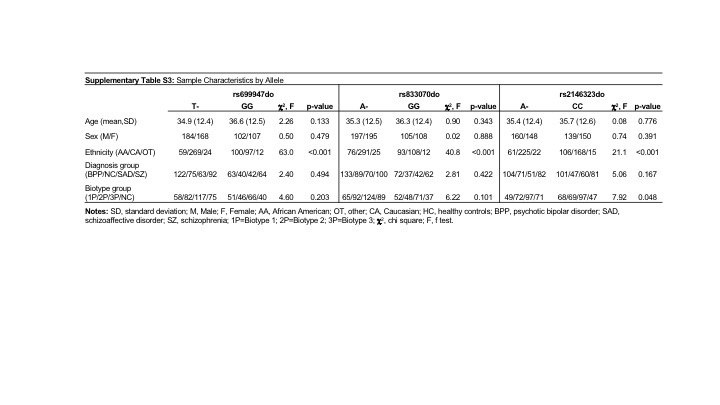

Supplement: Supplementary file 3 — Supplementary Table 3 [file 41398_2018_271_MOESM3_ESM.jpg]
