# Supplementary material for: VEGFA GENE variation influences hallucinations and frontotemporal morphology in psychotic disorders: a B-SNIP study
Source: Transl Psychiatry. 2018 Oct 11;8:215. doi: 10.1038/s41398-018-0271-y (PMC6181939; doi:10.1038/s41398-018-0271-y)
Supplement: Supplementary file 4 — Supplemental tables [file 41398_2018_271_MOESM4_ESM.pptx]

## Slide 1
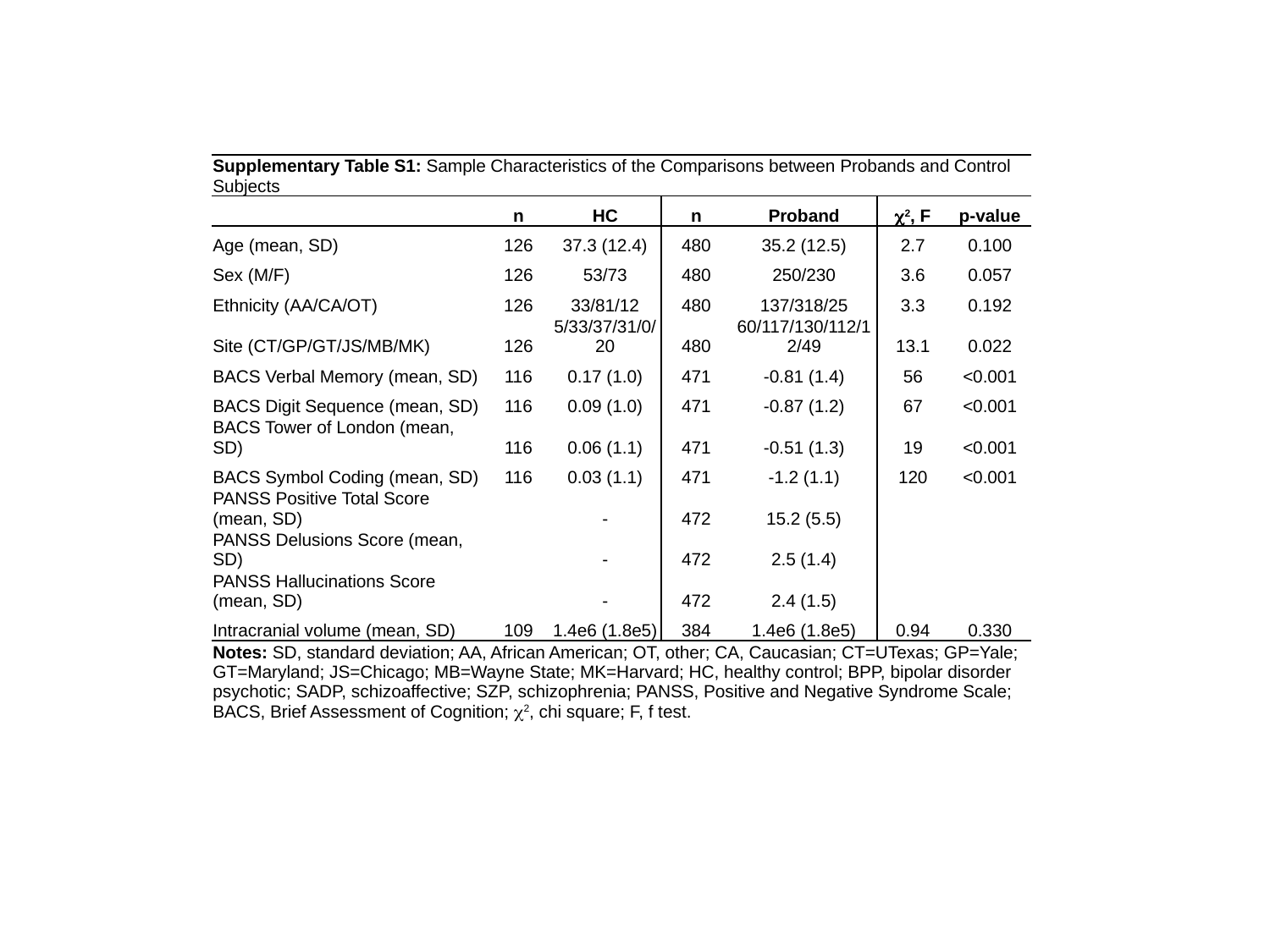

| Supplementary Table S1: Sample Characteristics of the Comparisons between Probands and Control Subjects | | | | | | |
| --- | --- | --- | --- | --- | --- | --- |
| | n | HC | n | Proband | c2, F | p-value |
| Age (mean, SD) | 126 | 37.3 (12.4) | 480 | 35.2 (12.5) | 2.7 | 0.100 |
| Sex (M/F) | 126 | 53/73 | 480 | 250/230 | 3.6 | 0.057 |
| Ethnicity (AA/CA/OT) | 126 | 33/81/12 | 480 | 137/318/25 | 3.3 | 0.192 |
| Site (CT/GP/GT/JS/MB/MK) | 126 | 5/33/37/31/0/20 | 480 | 60/117/130/112/12/49 | 13.1 | 0.022 |
| BACS Verbal Memory (mean, SD) | 116 | 0.17 (1.0) | 471 | -0.81 (1.4) | 56 | <0.001 |
| BACS Digit Sequence (mean, SD) | 116 | 0.09 (1.0) | 471 | -0.87 (1.2) | 67 | <0.001 |
| BACS Tower of London (mean, SD) | 116 | 0.06 (1.1) | 471 | -0.51 (1.3) | 19 | <0.001 |
| BACS Symbol Coding (mean, SD) | 116 | 0.03 (1.1) | 471 | -1.2 (1.1) | 120 | <0.001 |
| PANSS Positive Total Score (mean, SD) | | - | 472 | 15.2 (5.5) | | |
| PANSS Delusions Score (mean, SD) | | - | 472 | 2.5 (1.4) | | |
| PANSS Hallucinations Score (mean, SD) | | - | 472 | 2.4 (1.5) | | |
| Intracranial volume (mean, SD) | 109 | 1.4e6 (1.8e5) | 384 | 1.4e6 (1.8e5) | 0.94 | 0.330 |
| Notes: SD, standard deviation; AA, African American; OT, other; CA, Caucasian; CT=UTexas; GP=Yale; GT=Maryland; JS=Chicago; MB=Wayne State; MK=Harvard; HC, healthy control; BPP, bipolar disorder psychotic; SADP, schizoaffective; SZP, schizophrenia; PANSS, Positive and Negative Syndrome Scale; BACS, Brief Assessment of Cognition; c2, chi square; F, f test. | | | | | | |

## Slide 2
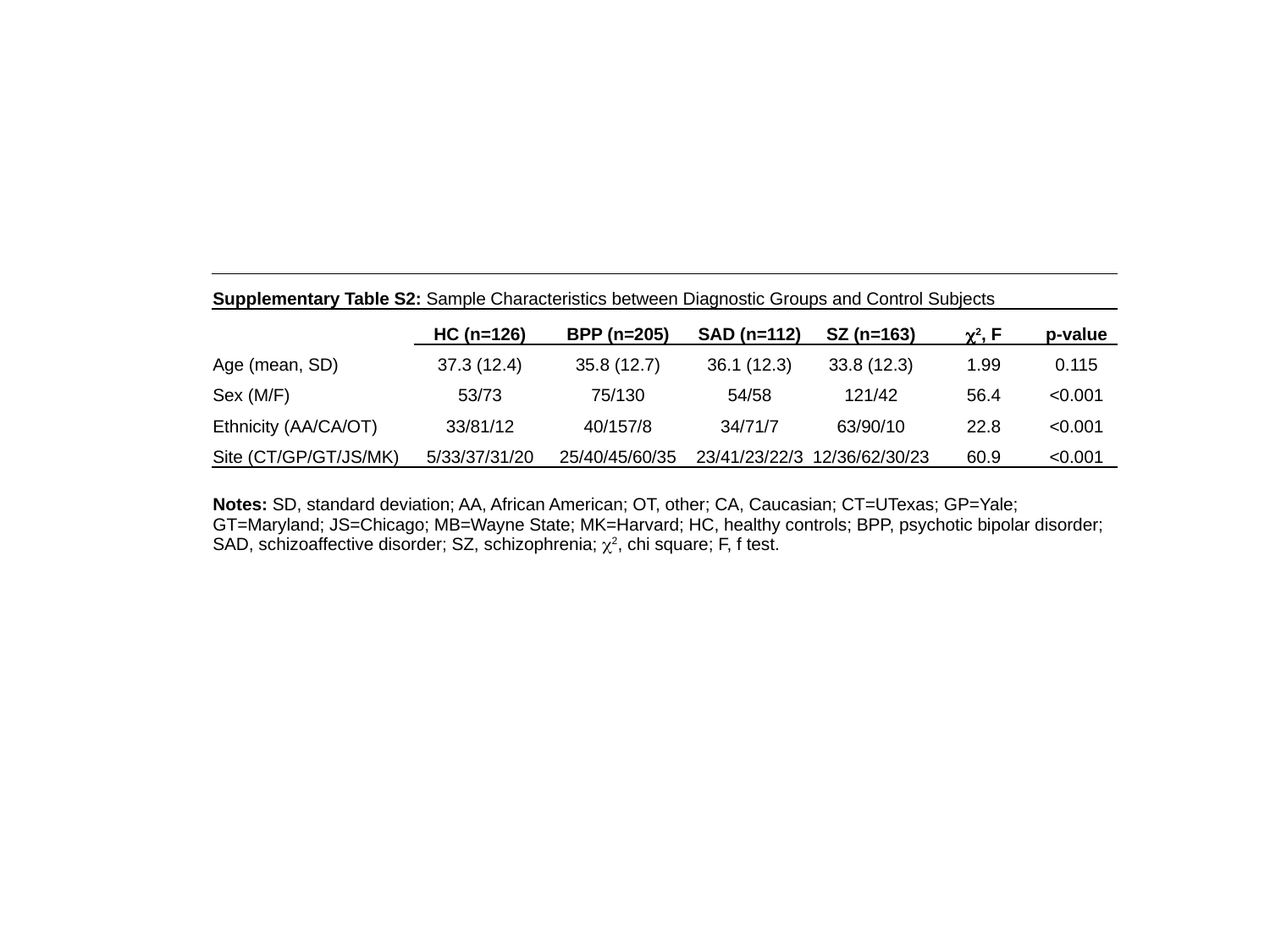

| Supplementary Table S2: Sample Characteristics between Diagnostic Groups and Control Subjects | | | | | | |
| --- | --- | --- | --- | --- | --- | --- |
| | HC (n=126) | BPP (n=205) | SAD (n=112) | SZ (n=163) | c2, F | p-value |
| Age (mean, SD) | 37.3 (12.4) | 35.8 (12.7) | 36.1 (12.3) | 33.8 (12.3) | 1.99 | 0.115 |
| Sex (M/F) | 53/73 | 75/130 | 54/58 | 121/42 | 56.4 | <0.001 |
| Ethnicity (AA/CA/OT) | 33/81/12 | 40/157/8 | 34/71/7 | 63/90/10 | 22.8 | <0.001 |
| Site (CT/GP/GT/JS/MK) | 5/33/37/31/20 | 25/40/45/60/35 | 23/41/23/22/3 | 12/36/62/30/23 | 60.9 | <0.001 |
| Notes: SD, standard deviation; AA, African American; OT, other; CA, Caucasian; CT=UTexas; GP=Yale; GT=Maryland; JS=Chicago; MB=Wayne State; MK=Harvard; HC, healthy controls; BPP, psychotic bipolar disorder; SAD, schizoaffective disorder; SZ, schizophrenia; c2, chi square; F, f test. | | | | | | |

## Slide 3
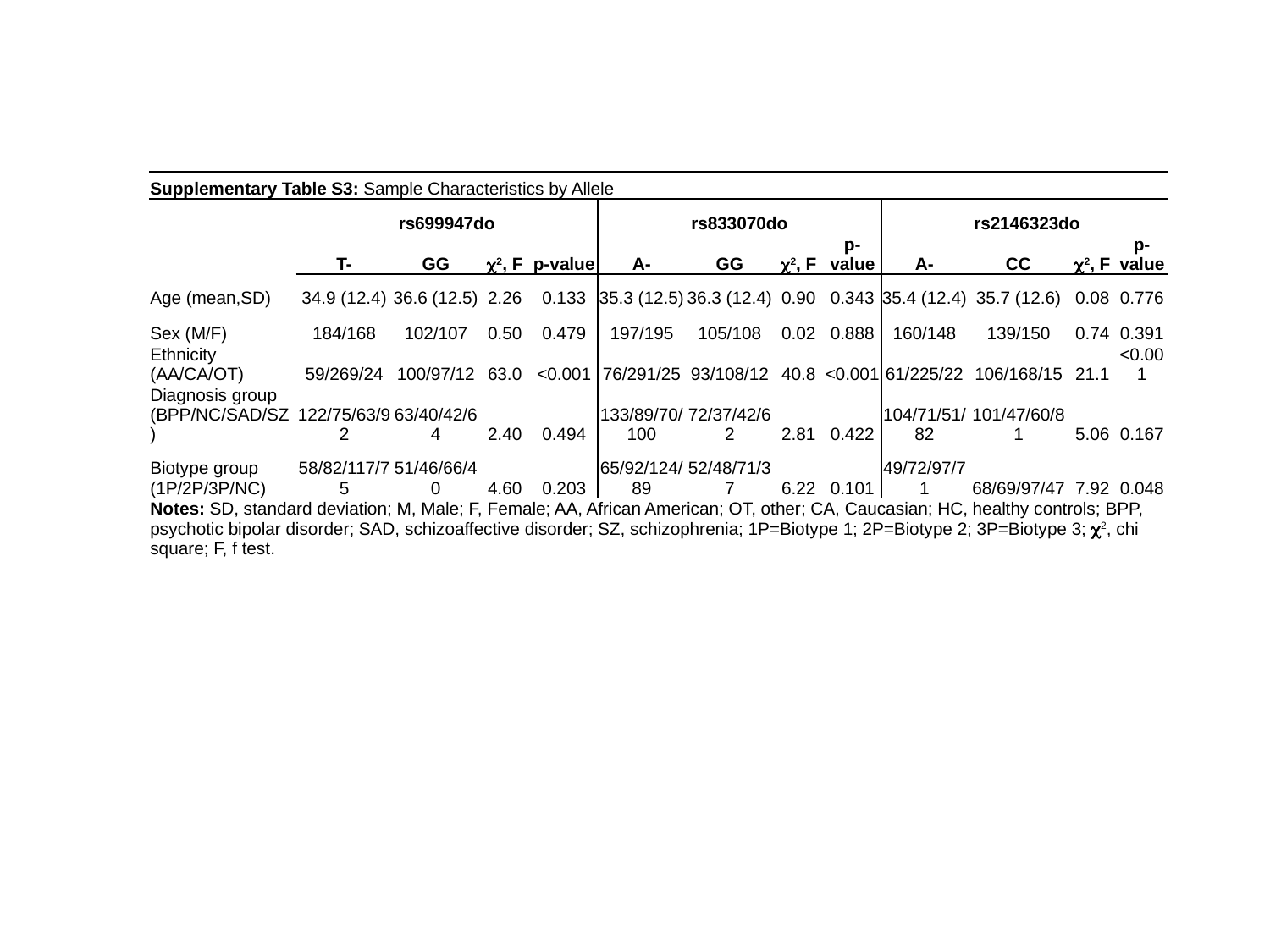

| Supplementary Table S3: Sample Characteristics by Allele | | | | | | | | | | | | |
| --- | --- | --- | --- | --- | --- | --- | --- | --- | --- | --- | --- | --- |
| | rs699947do | | | | rs833070do | | | | rs2146323do | | | |
| | T- | GG | c2, F | p-value | A- | GG | c2, F | p-value | A- | CC | c2, F | p-value |
| Age (mean,SD) | 34.9 (12.4) | 36.6 (12.5) | 2.26 | 0.133 | 35.3 (12.5) | 36.3 (12.4) | 0.90 | 0.343 | 35.4 (12.4) | 35.7 (12.6) | 0.08 | 0.776 |
| Sex (M/F) | 184/168 | 102/107 | 0.50 | 0.479 | 197/195 | 105/108 | 0.02 | 0.888 | 160/148 | 139/150 | 0.74 | 0.391 |
| Ethnicity (AA/CA/OT) | 59/269/24 | 100/97/12 | 63.0 | <0.001 | 76/291/25 | 93/108/12 | 40.8 | <0.001 | 61/225/22 | 106/168/15 | 21.1 | <0.001 |
| Diagnosis group (BPP/NC/SAD/SZ) | 122/75/63/92 | 63/40/42/64 | 2.40 | 0.494 | 133/89/70/100 | 72/37/42/62 | 2.81 | 0.422 | 104/71/51/82 | 101/47/60/81 | 5.06 | 0.167 |
| Biotype group (1P/2P/3P/NC) | 58/82/117/75 | 51/46/66/40 | 4.60 | 0.203 | 65/92/124/89 | 52/48/71/37 | 6.22 | 0.101 | 49/72/97/71 | 68/69/97/47 | 7.92 | 0.048 |
| Notes: SD, standard deviation; M, Male; F, Female; AA, African American; OT, other; CA, Caucasian; HC, healthy controls; BPP, psychotic bipolar disorder; SAD, schizoaffective disorder; SZ, schizophrenia; 1P=Biotype 1; 2P=Biotype 2; 3P=Biotype 3; c2, chi square; F, f test. | | | | | | | | | | | | |
